# Effects of Cultivar and Process Variables on Dynamic-Mechanical and Sensorial Behavior of Value-Added Grape-Based Smoothies

**DOI:** 10.3390/molecules171011421

**Published:** 2012-09-26

**Authors:** Antonietta Baiano, Marcella Mastromatteo, Matteo Alessandro del Nobile

**Affiliations:** Dipartimento di Scienze Agrarie, degli Alimenti e dell’Ambiente, Via Napoli, 25-71100 Foggia, Italy

**Keywords:** antioxidants, rheological properties, process variables, sensory properties, table grape

## Abstract

The effects of either cooking temperature (45, 80, and 100 °C) or inclusion of seed particles on the dynamic-mechanical and sensorial properties of value-added Crimson seedless, Black Pearl, or Baresana grape-based smoothies were studied. The inclusion of seed particles resulted in significant increases of the phenolic content, both in Black Pearl and Baresana, but it did not affect in a negative way the sensorial characteristics of smoothies whereas it caused an increase of the viscoelastic behavior of Black Pearl and a slight decrease in Baresana grape-based smoothies. In particular, the investigated rheological parameters were the loss and storage modulus. Moreover, the loss tangent value (the ratio between loss and storage modulus) remained unchanged, indicating a pseudoplastic behavior of all samples, independent on the process conditions. The smoothies produced from Crimson grapes at the intermediate temperature (80 °C) showed sensorial and rheological characteristics similar to those manufactured at 45 °C and better than those manufactured at 100 °C.

## 1. Introduction

A smoothie or purée is a thick, moist form of cooked vegetables, fruits, *etc*., ground, pressed, blended, and/or sieved to the consistency of a paste. It has been conceived as a valuable, ready-to-eat, and pleasant alternative to fresh fruits for who don’t like them, for kids and old people having difficulty in mastication, but also for people disliking food preparation operations such as washing, peeling, cutting, and destoning necessary to prepare a fruit meal. A smoothie can be eaten alone, or as a topping product, and also spread on a slice of bread, to supply a nourishing breakfast or snack. Table grapes are not widely used in smoothie-making, but can be considered a good raw material since they are rich in phytochemicals such as simple phenols (hydroxybenzoic and hydroxycinnamic acids), flavonoids (flavonols, flavanols, and anthocyanins), stilbenes (mainly, resveratrol), and proanthocyanidins [1,2]. To increase the intake of phytochemicals, the food industry is oriented to the production of functional products. The definition of “functional foods” is complex and continuously modified but it includes natural or processed foods containing known biologically-active compounds that when present in defined quantitative and qualitative amounts provides a health benefit. The term “value-added” refers to foods whose incremental value is due to their differences from similar products concerning geographical origin, food safety or also functionality.

In order to produce value-added grape-based smoothies, several strategies can be applied. For example, since phenolic compounds are distributed differently among the various parts of berries (the total extractable phenolics are present at only about 10% or less in pulp, 60%–70% in the seeds, and 28%–35% in the skin) [3], seeds and peels that are generally removed during processing [4], can be recovered, ground and re-added to the liquid portion. Nevertheless, the effects of the fibre particles and of the phenolic fraction on the dynamic-mechanical and sensory properties cannot be neglected. Another strategy can be represented by the combination of temperatures and various degrees of vacuum during cooking/concentration of the fruit purée. Ferracane *et al*. [5] observed an increase of antioxidant concentration during cooking of artichokes as an effect of matrix softening and consequent increased extractability of those compounds. Surely, further investigations on the effects of cooking on total phenolics as well as individual phenolic compounds are needed [6]. Furthermore, the effects of temperatures on the dynamic-mechanical and sensory properties (formation of lumps) should also be objects of study.

Sensory analysis in combination with mechanical measurements could represent the smoothie quality more accurately. The smoothie is a viscoelastic food material that exhibits both solid-like and fluid-like behavior. The texture of a smoothie has to provide a balance between desired mechanical stability (for storage and handling) and desired instability (to elicit a specific texture attribute during spreading over a piece of bread or during mastication) [7]. Rheological properties are useful in determining ingredient functionality in the product development, quality control, and correlation of food texture to sensory attributes [8,9]. The rheological behavior of smoothies has been widely studied [10,11,12,13]. It has been established that the rheological properties of smoothies are mainly affected by the amount and type of sugar added, proportion and kind of gelling agent used, fruit pulp content, and processing temperature.

Crimson seedless, Black Pearl, and Baresana were the table grape cultivars chosen for this research whose main aim was to investigate and contribute to knowledge of the effects of process variables such as cooking temperature and inclusion of peel and seed particles on the dynamic-mechanical and sensorial properties of value-added table grape-based smoothies.

## 2. Results and Discussion

### 2.1. Phenolic Content

**Figure 1:**
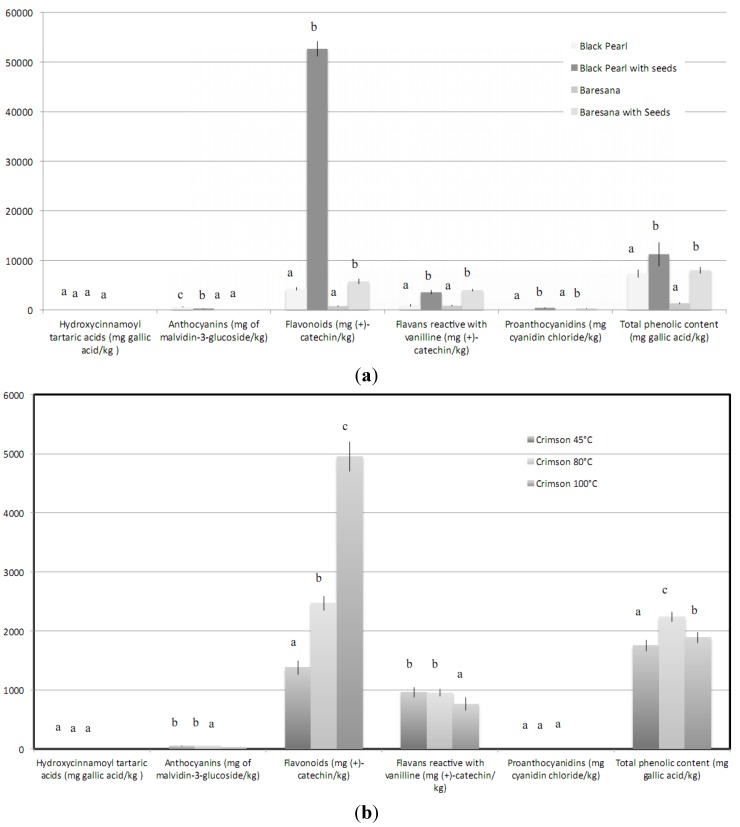
Phenolic composition of (**a**) Black Pearl and Baresana (**b**) Crimson grape-based smoothies. The results are expressed on a dry matter basis. Within each cultivar, different letters indicate significant differences at *p* < 0.05.

It is opportune to introduce this section by saying that the effects of jam and similar processing on phenolic components and antioxidant activity were evaluated by a few researchers [14]. In order to produce added-value smoothies, namely grape-based products rich in antioxidant compounds, the first aim was to quantify the influence of processing on the phenolic fraction. The effects of inclusion of seed particles on the phenolic fractions of grape smoothies are shown in Figure 1a. Hydroxycinnamoyl tartaric acids were not detected since they were rapidly oxidized by polyphenoloxidase during the early stages of processing. Concerning Black Pearl smoothies, those enriched with seed particles showed the lowest anthocyanin content as a consequence of the addition of a noticeable mass whose contribution of this class of phenolics was null. Flavonoids, flavans reactive with vanillin, proanthocyanidins, and the total phenolic contents obviously increased with the enrichment. The increases of phenolics shown by the enriched smoothies with respect to the samples produced without inclusion of seed particles were higher in the case of products derived from Baresana grapes, whereas the absolute phenolic concentrations of enriched Baresana smoothies were lower than those produced with Black Pearl grapes. In particular, the increase of the total phenolic fraction obtained through the inclusion of seed particles was equal to 53 and 491%, in Black Pearl and Baresana smoothies, respectively. These data confirm the utility of the addition of grape seeds. This represents an example of process simplification since it would make unnecessary the preliminary extraction of phenolic compounds. With regard to Crimson smoothies (Figure 1b) neither hydroxycinnamoyl tartaric acids nor proanthocyanidins were detected. The classes of anthocyanins and flavans reactive with vanillin were the most sensitive to heating, since their amounts were dramatically reduced in going from 80 to 100 °C, whereas flavonoids increased with temperature due to the already known increase of the ratio of free-to-bound compounds. The total phenolic content was the highest in samples produced from grapes cooked at 80 °C, whereas samples treated at 45 and 100 °C did not differ statistically from each other. These results could seem contradictory; nevertheless, they could be explained considering that cooking conditions can alter the free and bound phenolic content as result of the transformation or degradation that takes place during processing. It was also to take into account that the results deriving from the application of the Folin-Ciocalteau method could be altered by a series of interferences that, in grape derivatives are mainly represented by sugars and sulfur dioxide. Other interferences can become really important in the determination of total flavonoids when extracted with hydroalcoholic SO_2_-rich solvent from different matrices from wines [15]. They concern substances with λ_max_ at 280 nm that would seem to react with other phenolics modifying the absorption at 280 nm. Concerning the effects of jam processing, the results obtained by the various researchers include a wide range of states although any of them concerned the grape jam processing but the production of jams from other types of berry. Häkkinen *et al*. [16] highlighted that only a small portion of flavonols was lost during production of strawberry jam, whereas Amakura *et al*. [17] showed that total phenolics were preserved during blackberry jam processing. Howard *et al*. [18] found that blueberry jam processing resulted in losses of anthocyanins, procyanidins, chlorogenic acid, and antioxidant activity but flavanols well retained. The processing of raspberry jams determined a slightly flavanol decrease and a strong increase of the free ellagic acid whereas the ellagic acid derivatives remained quite stable [19]. In strawberry, sweet and sour cherry low sugar jam, the main losses were recorded for monomeric anthocyanins (92%–93%), vitamin C content (54%–78%), antioxidant activity (30%–41%), and total phenolic content (25%–43%) [20].

### 2.2. Sensorial Aspects

Once the effects of processing conditions on the value-added smoothies antioxidant content were assessed, it was necessary to evaluate their influence from a sensory point of view. Concerning the inclusion of seed particles, it contributed to the smoothie phenolic content by supplying flavonoids and proanthocyanidins (the main grape-derived tannins), formed by the polymerization of the flavan-3-ol monomers catechin and epicatechin. These subunits form chains of varying length and this is just the main variable, together with the nature of the individual subunits that compose them, characterizing their sensorial properties. Grape seed tannins are more astringent that skin tannins since they are generally constituted by a greater number of monomers and, furthermore, have a different structure based on galloyl esters [21]. Concerning the sensorial properties of Black Pearl and Baresana grape-based smoothies, the main effect of the inclusion of seed particles was the reduction of the mellow mouthfeel of smoothies caused by the presence of fibres. The latter was significantly perceived by judges (Table 1) since they increased the product grittiness mouthfeel but only in the case of Baresana they caused a significant increase of the astringency. In any case, the seed particles didn’t significantly affect the overall likeability score of the enriched products. These results were very interesting, since they suggest it is possible to create value-added foods without any significant detrimental effects on the product acceptability.

**Table 1:** Results of the sensorial analysis of the functional table grape-based smoothies.

Cultivar	Process variables	Presence of seeds parts	Presence of lumps	Astringency	Overall Likeability Score
Black Pearl	Without seed enrichment	0.0 ± 0.0 ^a^	-	1.75 ± 1.71 ^a^	2.6 ± 1.52 ^a^
	With seed enrichment	2.0 ± 1.58 ^b^	-	1.8 ± 1.10 ^a^	3.8 ± 0.84 ^a^
Baresana	Without seed enrichment	0.0 ± 0.0 ^a^	-	0.75 ± 0.96 ^a^	4.0 ± 2.0 ^a^
	With seed enrichment	2.2 ± 0.84 ^b^	-	2.0 ^a^ ± 1.22 ^b^	2.8 ± 1.64 ^a^
Crimson seedless	45 °C	-	1.2 ± 2.17 ^a^	-	3.6 ± 1.52 ^a^
	80 °C	-	0.8 ± 1.79 ^a^	-	4.0 ± 1.73 ^a^
	100 °C	-	1.2 ± 1.79 ^a^	-	2.6 ± 2.19 ^a^

In the columns, within each cultivar, different letters indicate significant differences at *p* < 0.05.

The main detrimental effect that should be avoided during smoothy or puree production is the formation of lumps due to the effects of cooking temperatures on water evaporation that leave the fruit pectins dehydrated. In order to prevent their formation, the heating process should be mild, thus allowing the formation of a uniform viscous solution. The lowest presence of lumps was detected on Crimson grape-based smoothies cooked at the intermediate temperature although that value did not differ significant from the other smoothies. Consistent with this result, judges expressed their highest overall liking for the same samples but, also in this case, the statistical differences with respect to the smoothies produced at 45 and 100 °C were not significant.

### 2.3. Rheological Aspects

Dynamic rheological tests were carried out on the selected value-added grape-based smoothies (Table 2). Results shown that the storage modulus, G’, was greater than the loss modulus, G”, at any given point in the frequency sweep tests (data not shown). This indicated a dominant contribution of the elastic component to the viscoelasticity of the investigated smoothies, behavior typical for a viscoelastic solid. This means that the attractive forces become dominant due to the strong hydrogen bond and hydrophobic association [7]. Similar data have been recorded for different food systems such as ovalbumin gel [22], jam and yoghurt [12], starch gel [23], and lactoglobulin gel [24].

**Table 2:** Rheological parameters of the functional table grape-based smoothies.

Cultivar	Process variables	G’	G”	tanδ
Black Pearl	Without seed enrichment	1,302.81 ± 145.5 ^a^	419.60 ± 45.29 ^a^	0.32 ± 0.002 ^a^
	With seed enrichment	18,081.51 ± 5,819.02 ^b^	6,977.30 ± 241.41 ^b^	0.46 ± 0.029 ^b^
Baresana	Without seed enrichment	238.80 ± 69.31 ^a^	77.38 ± 21.80 ^a^	0.32 ± 0.002 ^b^
	With seed enrichment	1,762.25 ± 274.89 ^b^	511.72 ± 95.30 ^b^	0.28 ± 0.009 ^a^
Crimson	45 °C	1,271.26 ± 410.14 ^b^	290.51 ± 104.31 ^b^	0.29 ± 0.003 ^a^
	80 °C	824.08 ± 115.13 ^b^	293.78 ± 88.83 ^b^	0.29 ± 0.007 ^a^
	100 °C	73.37 ± 10.18 ^a^	21.77 ± 2.93 ^a^	0.29 ± 0.001 ^a^

In column, within each cultivar, different letters indicate significant differences at *p* < 0.05.

Concerning the effect of processing on the rheological parameters of investigated smoothies, the results highlighted that for the cultivar Black Pearl and Baresana the inclusion of seeds led to significant increases of both the moduli with respect to the smoothies whose manufacturing did not include enrichment. This behavior is related to a more structured and elastic sample. Igual, Contreras and Martínez-Navarrete [25] found that the incorporation into grapefruit jam of fibre, *i.e*., by addition of apple and orange, increased the storage and loss modules, thus contributing to the viscoelasticity of the system. The enrichment caused a significant increase of tanδ (ratio between loss and storage modulus) in the case of Black Pearl and a slight but significant decrease for Baresana smoothies. The tanδ value, also known as loss tangent, is a direct measure of the relative importance of viscous and elastic effects in the sample. For all the considered samples, tanδ was lower than 1, thus indicating a gel-like behavior [26] (Table 2). Regarding the Crimson-based smoothies, the increase of cooking temperatures from 45 to 80 °C did not significantly affect the two moduli, whereas it caused their significant decrease when the temperature rose from 80 to 100 °C with the typical viscoelastic behavior of a weak gel. In fact, during concentration, part of the water contained in the fruit evaporated whereby tissues become softer, absorbed part of the water and released pectin and acids [27]. The ratio between loss and storage modulus remained unchanged, indicating that loss tangent was independent of the process conditions. Guerrero and Alzamora [28] observed the same pseudoplastic behavior for the analyzed fruit purèes. They also found that the temperature slightly affected this behavior. Moreover, Saravacos [29] also reported the limited variation of the flow index value with some processing variables in fruit purèes.

On the other hand, Chin, Chan, Yusof, Chuah and Talib [30] found that the pseudoplastic behavior of pummelo juice increased with concentration of juice while the effect of temperature seems to reduce its pseudoplasticity at each concentration level. Moreover, the consistency coefficient decreased with temperature, but increased with concentration of pummelo juice concentrates. This phenomenon of viscosity increase with total soluble solids content and decrease in viscosity with temperature is consistent with many other studies, e.g., in guava puree by Vitali and Rao [31], apricot puree by Duran and Costell [32], clarified peach juice by Ibarz, Gonzalez, Esplugas and Vicente [33], orange juice by Ibarz, Gonzalez and Esplugas [34], clarified apple juice by Constenla, Lozano and Crapiste [35], and grape juice by Bayindirli [36].

### 2.4. Correlation between Sensorial and Rheological Characteristics

Table 3 reports the correlations between sensorial and rheological properties. The overall likeability score of Black Pearl and Baresana smoothies was positively, but not highly correlated to the three rheological parameters, whereas the sensorial attribute “Presence of seed parts” was negatively correlated to G’, G”, and tanδ. This latter result is in contrast with what found by means of the instrumental analysis. In fact, the effect of the seeds concentration on the rheological instrumental parameters showed a positive correlation. Most probably, it was due to the fact that, the sensorial parameter (presence of seed parts) is less sensitive to small variations whereas the instrumental parameters (G’ and G”) are more objective because all elements of subjectivity were removed from the testing.

**Table 3:** Correlation results between sensorial attributes and rheological parameters of the functional table grape-based smoothies.

Black Pearl/Baresana	G’	G”	tanδ	Presence of seeds parts	Overall Likeability Score
G’	1.000000	-	-	-	-
G”	0.999804	1.000000	-	-	-
tand	0.955220	0.960616	1.000000	-	-
Presence of seed particles	−0.514591	−0.529541	−0.670555	1.000000	-
Overall Liking Score	0.413307	0.427125	0.540992	−0.976354	1.000000
Crimson	G’	G”	tan	Presence of lumps	Overall Liking Score
G’	1.000000	-	-	-	-
G”	0.925361	1.000000	-	-	-
tand	−0.989468	−0.860741	1.000000	-	-
Presence of lumps	−0.144752	−0.509043	0.000000	1.000000	-
Overall Liking Score	0.790378	0.963621	−0.693375	−0.720577	1.000000

The overall likeability score of Crimson smoothies was highly and positively correlated to G’ and G” and negatively correlated to tanδ. This result highlighted that the overall liking score increased with the increase of both the storage and loss modulus that is when the smoothies had a more elastic and structured texture. On the other hand, this means that the smoothies were more pleasant when their behavior approached to that of a pseudoplastic material, that is when tanδ value decreased.

### 2.5. Classification of Samples Based On Formulations or Processing Conditions

**Figure 2:**
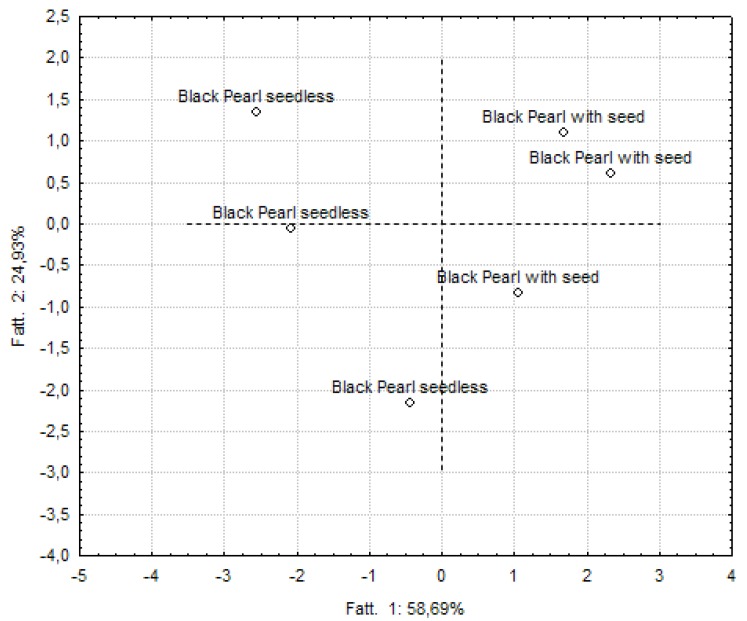
Principal component analysis applied to Black Pearl grape-based smoothies.

**Figure 3:**
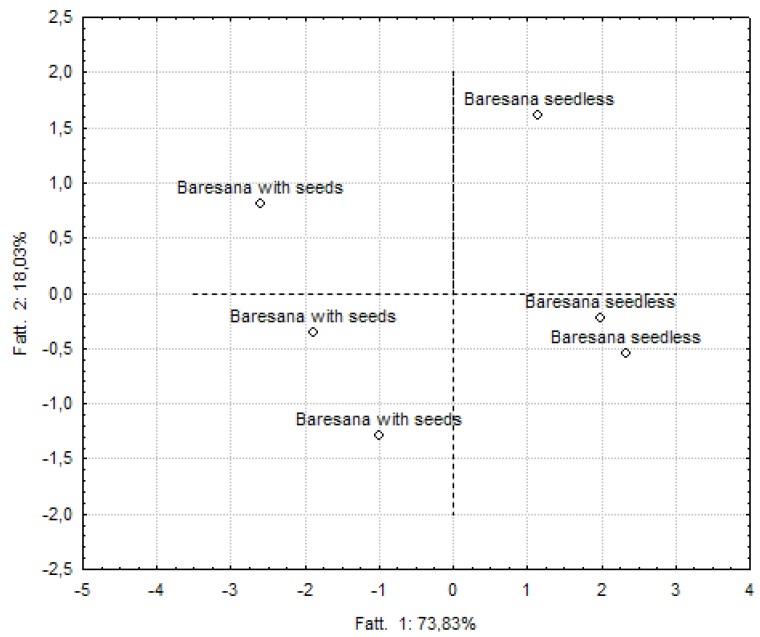
Principal component analysis applied to Baresana grape-based smoothies.

**Figure 4:**
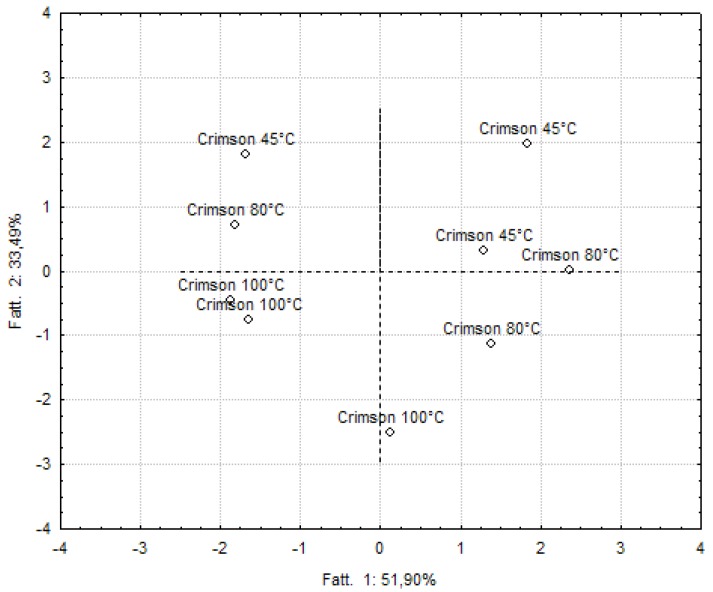
Principal component analysis applied to Crimson grape-based smoothies.

Separately for each cultivar, PCA was employed to visualize groupings of samples on the basis of processing operations. Loading plots obtained from PCA of all the parameters are represented in Figure 2, Figure 3, Figure 4 for Black Pearl, Baresana, and Crimson smoothies, respectively. With regard to Black Pearl, the first two factors of the statistical analysis explained over 83% of the variance and all the indices were useful variables for an attempt of separation of the enriched smoothies from the others. The resulting graphs allowed to group smoothies obtained without inclusion of seed particles in the region of the planes characterized by negative values of the factor 1 and enriched samples in the part of the plane characterized by positive values of the same factor.

A clear separation between enriched and non-enriched smoothies was also scored for smoothies manufactured from Baresana, with about 92% of the variance explained by the first two factors. Concerning Crimson grape-based smoothies, the first two factors explained 85.39%, but the resulting graphs did not allow a clear separation of samples on the basis of the cooking temperature.

## 3. Experimental

### 3.1. Grape Production

Crimson seedless table grape represents the preferred red seedless for supermarkets worldwide due to its sweet neutral juicy flavour and elongated berries that are light red in colour, crisp and firm in texture and grows in large triangle clusters. Black Pearl is a recent black grape cultivar, characterized by large bunches, crispy flesh, neutral flavour, pruinose and thick skins, and 1 or 2 seeds per berry. Baresana is an ancient and vigorous cultivar of white grapes, coming from Apulia, in the South of Italy. It is characterized by bunches of medium or big size, round and regular berries, skins of a golden yellow colour and not very pruinose, and crispy pulps with a sweet taste.

Trials were conducted on table grapes deriving from a vineyard in the countryside of Turi (Apulia region, Southern Italy). Soil and weather conditions were thus the same for all. The site is located in a hilly area at about 250 m above sea level and has a continental climate, characterized by a wide temperature range.

The “140 Ru” rootstock was planted in 2001 and, one year later, Baresana, Black Pearl, and Crimson seedless cultivar was grafted on it. The plant density of Crimson seedless vines was 1,890 vines/ha, spaced at 2.3 m (within rows) by 2.3 m (between rows). Black Pearl vines were spaced at 2.5 m (within rows) by 2.5 m (between rows) (1,600 vines × hectar). Baresana vines were spaced at 1 m (within rows) by 2.5 m (between rows) (4,000 vines × hectar).

Crimson seedless and Black Pearl grapevines were trained to the “double tendone” trellis system, a training system widely used in Southern Italy. Baresana was trained to Guyot system and 10 buds for two canes pruned (20 buds per plant).

The experimental vineyards were covered by high density polyethylene (HDPE) nets since these nets cover about 40% of the “Tendone” table grape vineyard surface in Apulia and 80% of the “Tendone” table grape vineyards in the South of Italy in order to protect the leaves and the grapes from hail and wind. The characteristics of the nets (5/4 Anti-hail net-Kristal, Retilplast, Salerno, Italy) used in this work are the following: english texture, transparent wires of 30 µm diameter, 75% porosity, and a shade factor of 14%.

The vineyards were irrigated by a localized irrigation system consisting into two 8-L h^−1^ pressure compensated droppers per vine, placed at a distance of 0.70 m from each other, in order to counterbalance the evapotranspiration ratio taking into account the rainfall.

Grapes were harvested at the same harvest time (from 20th to 30th September). Only clusters placed on the basal position of the shoots, in the median part of the fruit cane, were picked. The samples, placed into fruit boxes (4.5–5 kg per box) were immediately delivered to the laboratory and processed within 24 hours.

### 3.2. Smoothie Processing

Bunches were selected, hand-destemmed and any berries affected by moulds and rots discarded. The selected berries were washed under tap water, drained, squashed, and passed through a pulper (hole diameter 1 mm) to remove coarse fibres, stems, peel particles, and, if necessary, seeds.

In the case of Crimson seedless, three different cooking conditions were applied: a traditional cooking at 100 °C and atmospheric pressure and two cooking under vacuum conditions performed at 80 and 45 °C, respectively, to check the effects of heating on physicochemical and sensory characteristics and antioxidants. In all the pressure and temperature conditions, cooking was carried out until soluble solid concentration was around 54 °Brix (54.2 ± 0.1, 54.3 ± 0.8, 53.6 ± 1.1 for smoothies manufactured at 45, 80, and 100 °C, respectively).

In the case of Black Pearl and Baresana, after the passage through the pulper, the liquid portion was divided into two parts. One of the parts was added with the corresponding part of seeds previously grounded through a domestic grinding machine and homogenized at 3,000 *g* for 120 s in order to obtain particles lower than 2 mm. Then, smoothies with and without enrichment were cooked at 45 °C under vacuum conditions. The final soluble solid contents of Black Pearl smoothies were 57.59 ± 0.31 (without addition of seed particles) and 33.91 ± 2.88 (with addition of seed particles) °Brix. In the case of Baresana smoothies, the final soluble solid contents were 56.01 ± 0.34 (without addition of seed particles) and 51.44 ± 0.20 (with addition of seed particles) °Brix.

Immediately after evaporation/cooking, purees were filled into 340-mL jars, heated at 90 °C for 20 min and cooled to room temperature. Purees were stored at 15–20 °C and analyzed two weeks after production.

### 3.3. Extraction and Determination of the Phenolic Content

Extraction of the phenolic fractions was made according to Di Stefano and Cravero [37]. The puree samples were weighted and added with a solution of ethanol-water-37% hydrogen chloride (70:30:1). After 24 hours under dark conditions, the mixtures were filtered and immediately analyzed or stored at −18 °C.

The total phenolic content was measured at 765 nm using an UV-visible spectrophotometer (Varian Cary 50 SCAN, Palo Alto, CA, USA) according to the Folin-Ciocalteu method as reported by Singleton and Rossi [38]. Results were expressed as gallic acid equivalents (mg kg^−1^ of dry matter). A calibration line was built on the basis of solutions at known and increasing concentrations of gallic acid (ExtraSynthese, Genay, France). The total anthocyanin contents were measured at 540 nm and expressed as mg of malvidin-3-glucoside per kg of dry matter. The total flavonoids were calculated on the basis of their absorbance at 280 nm and the results expressed as mg of catechin per kg of dry matter. Flavans, expressed as mg of catechin per kg of dry matter, were determined using the vanillin assay at 500 nm. Proanthocyanidins were measured at 532 nm after acid hydrolysis at high temperature and expressed as mg of cyanidin chloride per kg of dry matter. The hydroxycinnamoyl tartaric acids were expressed as mg of caffeic acid per kg of dry pulp and measured on the basis of their absorbance at 320 nm. At least three repetitions of the spectrophotometric analyses were performed for each smoothie sample.

### 3.4. Sensorial Analysis

A panel of 15 trained people performed a quantitative descriptive analysis (QDA) of the smoothies. A profile sheet including four visual (brightness, colour uniformity, typical colour of grape, browning/anomalous colour), one olfactory (grape fruity flavour) and five taste/tactile (presence of lumps or fibre particle, sweetness, acidity, astringency, grape fruity taste) attributes was submitted to them. The trained peolple were requested to express their judgment on a 7-point scale (from 0 to 6) and they were also requested to express an overall liking score (overall judgment on a 0 to 6 scale) since they were regular consumers of marmalades, jams, purees, and smoothies. Among the sensory attributes evaluated, those related to dynamic-mechanical aspects were the presence of lumps/fibre particles and the overall liking score. Attributes were evaluated on a 0 (very weak) to 6 (very intense) scale. Each session lasted 2 hours, whereas the time between sessions was 24 hours. Products were presented on white plastic plates and tasted at room temperature. The sensory sessions were conducted in a sensory room equipped with eight booths according to the ISO standard 8589 (2007). The temperature was kept at 24 ± 2 °C. Humidity values were in the 70 to 85% range.

### 3.5. Rheological Analyses

The dynamic-mechanical properties of the smoothie samples were studied using a controlled-strain rotational rheometer (ARES model, TA Instruments, New Castle, DE, USA) equipped with a force rebalance transducer (model 1K-FRTN1, 1–1,000 gcm, 200 rad s^−1^, 2–2,000 grams force) and parallel plates (superior plate diameter of 50 mm). A steady temperature was ensured with an accuracy of ±0.1 °C by means of a controlled fluid bath unit and an external thermostatic bath. To prevent water evaporation, a suitable cover tool sealing the top of the superior plate was used during testing. Storage modulus (G’), loss modulus (G”) and tanδ were determined in a frequency range of 0.01 to 10 Hz. The strain value was obtained by preliminary strain sweep oscillatory trials to determine the linear viscoelastic region. The strain sweep oscillatory tests were carried out at a frequency of 1 Hz and in a range of shear strain of 0.01 to 99%. All experiments were carried out at 25 °C. Three repetitions of the dynamic-mechanical experiments were performed for each smoothie sample.

### 3.6. Data Analysis

Each type of grape smoothies was produced in triplicate. For each repetition, the extractions of the phenolic compounds were carried out at least three times and analyses were performed at least in triplicate. All other analyses were performed at least in triplicate. The averages and the standard deviations were calculated using Excel software ver. 11.5.1 (Microsoft, Redmond, WA, USA). The Analysis of Variance (ANOVA) at *p* < 0.05 followed by the LSD test was applied to highlighted significant differences among samples. The Principal Component Analysis (PCA) was applied to separate sample on the cultivars and/or locations according to all the parameters investigated. The eigenvectors were adopted to explain the projection of the samples on the factor-plane. The data were autoscaled before analysis. All the statistical analyses were made by the software Statistica ver. 5.1 (Statsoft, Tulsa, OK, USA).

## 4. Conclusions

With regard to Black Pearl and Baresana cultivars, the grape-based smoothies with the higher phenolic contents were those obtained by addition of seed particles whereas, concerning the Crimson cultivar, the higher nutraceutical value was detected on smoothies manufactured at the intermediate cooking temperature. The processing that gave rise to the smoothies with the higher phenolic content did not worsen their sensorial characteristics. Concerning the rheological aspects, the enrichment with seed particles significantly increased both the storage and loss modulus with respect to the products without enrichment although the viscous behavior was predominant. With regards to Crimson smoothies, those obtained at the intermediate cooking temperature showed the same rheological behavior of the products manufactured at the lower temperature, whereas a further increase of temperature gave rise to smoothies with a weak gel structure.

## References

[1] Hermann K. (1989). Occurrence and content of hydroxycinnamic and hydroxybenzoic acid compounds in foods. Crit. Rev. Food Sci. Nutr..

[2] Iriti M., Rossoni M., Borgo M., Ferrara L., Faoro F. (2005). Induction of resistance to gray mold with benzothiadiazole modifies amino acids profile and increases proanthocyanidins in grape: Primary versus secondary metabolism. J. Agric. Food Chem..

[3] Shi J., Yu J., Pohorly J.E., Kakuda Y. (2003). Polyphenolics in grape seeds-biochemistry and functionality. J. Med. Food.

[4] Qian N. (2006). Fruit and vegetable smoothies, and its processing method. Faming Zhuanli Shenqing Gongkai Shuomingshu,.

[5] Ferracane R., Pellegrini N., Visconti A., Graziani G., Chiavaro E., Miglio C., Fogliano V. (2008). Effects of different cooking methods on antioxidant profile, antioxidant capacity, and physical characteristics of artichoke. J. Agric. Food Chem..

[6] Rickman J.C., Barrett D.M., Bruhn C.M. (2007). Nutritional comparison of fresh, frozen and canned fruits and vegetables. Part 1. Vitamins C and B and phenolic compounds. J. Sci. Food Agric..

[7] Basu S., Shivhare U.S., Singh T.V., Beniwal V.S. (2011). Rheological, textural and spectral characteristics of sorbitol substituted mango jam. J. Food Eng..

[8] Kokini J.L., Plutchok G.J. (1987). Viscoelastic properties of semisolid foods and their biopolymers components. Food Technol..

[9] Dervisi P., Lamb J., Zabetakis I. (2001). High pressure processing in jam manufacture: Effect on textural and colour properties. Food Chem..

[10] Carbonell E., Costell E., Duran L. (1991). Rheological behavior of sheared jams: Relation with fruit content. J. Texture Stud..

[11] Carbonell E., Costell E., Duran L. (1991). Rheological indices of fruit content in jams: Influence of formulation on time-dependent flow of sheared strawberry and peach jam. J. Texture Stud..

[12] Gabriele D., de Cindio B., D’Antona P. (2001). A weak gel model for foods. Rheol. Acta.

[13] lvarez E., Cancela M.A., Maceiras R. (2006). Effect of temperature on rheological properties of different jams. Int. J. Food Prop..

[14] Rababah T.M., Al-Mahasneh M.A., Kilani I., Yang W., Alhamad M.N., Ereifej K., Al-u’datt A. (2011). Effect of jam processing and storage on total phenolics, antioxidant activity, and anthocyanins of different fruits. J. Sci. Food Agric..

[15] Corona O., Squadrito M., Borsa D., di Stefano R. (2010). Behaviour of some compounds with λ_MAX_ at 280 nm in the determination of total flavonoids of grape skin extracts made from a hydroalcoholic SO_2_-rich solvent. Ital. J. Food Sci..

[16] Häkkinen S.H., Kärenlampi S.O., Mykkänen H.M., Törrönen A.R. (2000). Influence of domestic processing and storage on flavonol contents in berries. J. Agric. Food Chem..

[17] Amakura Y., Umino Y., Tsuji S., Tonogai Y. (2000). Influence of jam processing on the radical scavenging activity and phenolic content in berries. J. Agric. Food Chem..

[18] Howard L.R., Castrodale C., Brownmiller C., Mauromoustakos A. (2010). Jam processing and storage effects on blueberry polyphenolics and antioxidant capacity. J. Agric. Food Chem..

[19] Zafrilla P., Ferreres F., Tomás-Barberán F.A. (2001). Effect of processing and storage on the antioxidant ellagic acid derivatives and flavonoids of red raspberry (Rubus idaeus) jams. J. Agric. Food Chem..

[20] Poiana M.-A., Moigradean D., Dogaru D. (2011). Constantin mateescu, diana raba, iosif gergen Processing and storage impact on the antioxidant properties and color quality of some low sugar fruit jams. Rom. Biotechnol. Lett..

[21] Cheynier V., Andersen O.M., Markham K.R. (2006). Flavonoids in Wine. Flavonoids—Chemistry, Biochemistry and Applications.

[22] Nakamura K., Kiriyama M., Takada A., Maeda H., Nemoto N. (1997). Structure and dynamics of ovalbumin gel III. Solvent effect. Rheol. Acta.

[23] Rosalina I., Bhattacharya M. (2001). Flow curves, stress relaxation and creep measurements of starch gels. J. Texture Stud..

[24] Goncalves M.P., Sittikijyothin W., da Silva M.V., Lefebvre J. (2004). A study of the effect of locust bean gum on the rheological behaviour and microstructure of a b-lactoglobulin gel at pH 7. Rheol. Acta.

[25] Igual M., Contreras C., Martínez-Navarrete N. (2010). Non-conventional techniques to obtain grapefruit jam. Innovat. Food Sci. Emerg. Technol..

[26] Mounsey J.S., O’Riordan E.D. (2001). Characteristics of imitation cheese containing native starches. J. Food Sci..

[27] Castelló M.L., Heredia A., Domínguez E., Ortolá M.D., Tarrazó J. (2011). Influence of thermal treatment and storage on astringency and quality of a spreadable product from persimmon fruit. Food Chem..

[28] Guerrero S.N., Alzamora S.M.  (1998). Effect of pH, Temperature and Glucose Addition on Flow Behaviour of Fruit Purees: II. Peach, Papaya and Mango Purèes. J. Food Eng..

[29] Saravacos G.D. (1970). Effect of temperature on viscosity of fruit juices and purees. J. Food Sci..

[30] Chin N.L., Chan S.M., Yusof Y.A., Chuah T.G., Talib R.A. (2009). Modelling of rheological behaviour of pummelo juice concentrates using master-curve. J. Food Eng..

[31] Vitali A.A., Rao M.A. (1982). Flow behaviour of guava puree as a function of temperature and concentration. J. Texture Stud..

[32] Duran I., Costell E. (1982). Rheology of apricot puree: Characterisation of flow. J. Texture Stud..

[33] Ibarz A., Gonzalez C., Esplugas S., Vicente M. (1992). Rheology of clarified fruit juices I: Peach juices. J. Food Eng..

[34] Ibarz A., Gonzalez C., Esplugas S. (1994). Rheology of clarified fruit juices III: Orange juices. J. Food Eng..

[35] Constenla D.T., Lozano J.E., Crapiste G.H. (1989). Thermophysical properties of clarified apple juice as a function of concentration and temperature. J. Food Sci..

[36] Bayindirli L. (1993). Density and viscosity of grape juice as a function of concentration and temperature. J. Food Process. Pres..

[37] Di Stefano R., Cravero M.C. (1991). Metodi per lo studio dei polifenoli delle uve. Rivista di Viticoltura ed Enologia.

[38] Singleton V.L., Rossi J.A. (1965). Colorimetry of total phenolics with phosphomolybdic phosphotungstic acid reagents. Am. J. Enol. Vitic..

